# Multiple osteofibrous dysplasia combined with femoral fracture with proximal femur shepherd's crook and femoral pseudojoint formation: case report and literature review

**DOI:** 10.3389/fsurg.2025.1432735

**Published:** 2025-06-24

**Authors:** Zongpu Wang, Song Qin, Tienan Wang, Jianchuan Wang

**Affiliations:** ^1^ Department of Orthopedics, Affiliated Zhongshan Hospital of Dalian University, Dalian, China; ^2^ School of Mechanical Engineering, Dalian Jiaotong University, Dalian, China

**Keywords:** fibrous dysplasia, shepherd's crook deformity, femoral pseudojoint, fibula graft, surgical treatment

## Abstract

**Background:**

Osteofibrous dysplasia is a congenital, non-hereditary benign bone disease characterized by localized bone protrusion and replacement of normal bone cancellous by proliferating abnormal bone fibers. For this case, there is no unified treatment standard for internal fixation reconstruction or replacement, which is mainly based on comprehensive evaluation of each patient's clinical history and imaging findings.

**Case presentation:**

We report a case of systemic multiple osteofibrous dysplasia complicated by left femoral bone fracture, left proximal-femur shepherd's crook deformity, and femoral pseudojoint formation. According to the patient's previous medical history and admission imaging examination, large segments of the ipsilateral fibula were removed, the bone marrow cavity was implanted, locking plate screws were inserted through the fibula in the pulp cavity, and steel cables were added to enhance stability. After 1 year of follow-up, the fracture had healed, and the patient returned to the prefracture walking state with satisfactory clinical results.

**Conclusions:**

For rare cases of systemic multiple fibrous dysplasia combined with femoral fracture with proximal-femur shepherd's crook deformity and femoral pseudojoint formation, either internal fixation reconstruction or hip replacement, specific analysis should be performed to provide a reference for future clinical diagnosis and treatment of this disease.

## Introduction

Fibrous dysplasia of bones (FDB), also known as osteofibroma, is a congenital, nongenetic, benign bone disease characterized by limited bone protrusion and replacement of normal bone cancellous material by proliferating abnormal bone fibers. Wiel was the first to discover and report in 1922, followed by Albright in 1937 and 1938, who reported seven consecutive similar cases. In 1942, Lichten-Stein and Jaffe (1) first named the disease abnormal proliferation of bone fibers, and studies have reported that the disease accounts for approximately 7% of nonmalignant bone tumors, with a malignancy rate ranging from 2% to 3% (2, 3). The etiology of the disease is still unknown, though most scholars believe that it is caused by abnormal development of primitive mesenchymal tissues and abnormal proliferation of fibrous tissues in the bone. It can manifest as simple fibrous dysplasia (70%–80%), polyostotic fibrous dysplasia (20%–30%), or McCune-Albright syndrome (2%–3%) (4). First described by von Recklinghausen in 1891, the proximal femur is one of the most frequent sites of progressive inversion and curvature due to mechanical stress and repeated fractures, which is typical of shepherd's crook deformity, a deformity that develops through osteoporosis, persistent microfractures, and fracture repair; it is associated with limb pain, claudication, and fracture of the neck of the femur (5).

Studies have reported (6) many structural abnormalities of multiple bone fibers, such as multiple single-femur, multiple single-tibia or bilateral tibia and bilateral femur fractures. Currently, more rarely seen systemic multiple bone fiber anomalies may be combined with femur fracture with proximal-femur shepherd's crook deformity. A few cases of generalized multiple bone fiber anomalies combined with femoral fracture with proximal-femur shepherd's crook deformity and femoral pseudoarthrosis have been reported. We report a case of generalized multiple bone fiber dysplasia combined with femoral fracture with proximal-femur shepherd's crook deformity and femoral pseudoarthrosis, which, to the best of our knowledge, is uncommon in China and abroad. We hope that by describing this case and the treatment plan, we can provide clinicians with a better understanding of such cases and a reference experience for choosing treatments for this kind of disease in the future.

## Case presentation

**Figure 1:**
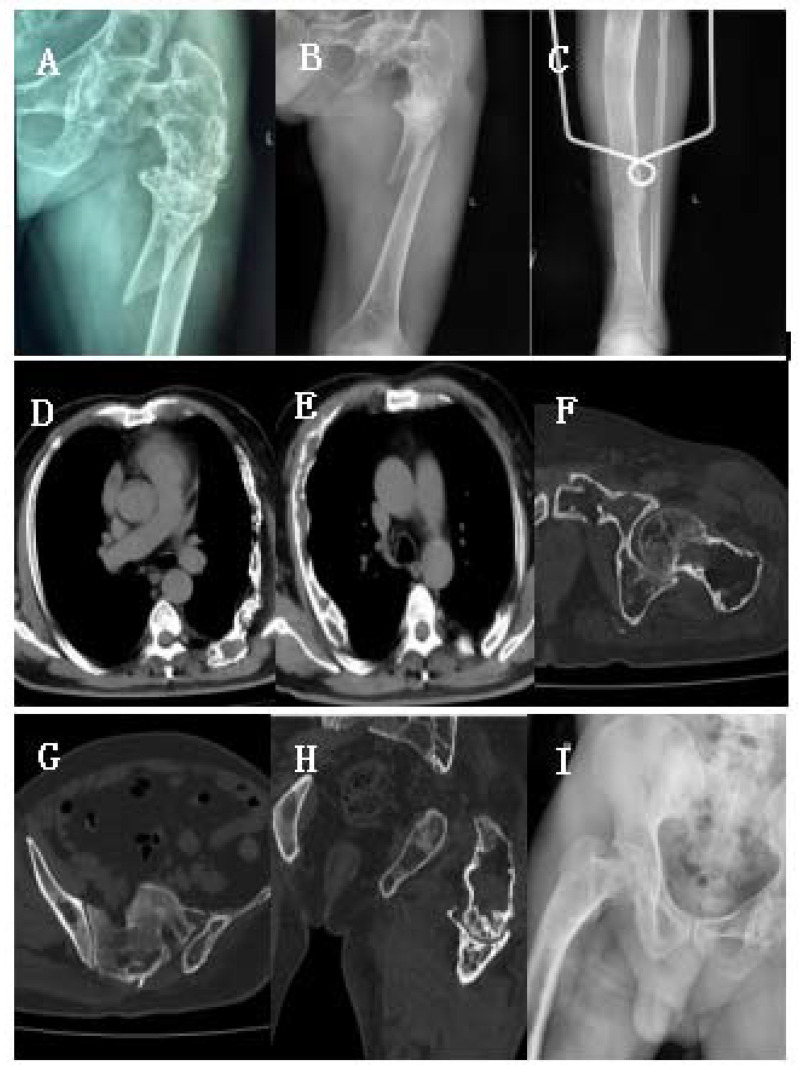
**(A–I)** Preoperative clinical imaging data. X-ray and CT showed fractures at the proximal end of the left femur, shepherd's crook deformity, and multiple fibrous dysplasia in the femur, ribs, acetabulum, and tibia.

A 44-year-old male fell accidentally while taking a shower at home and landed on his left hip. He immediately experienced severe pain in his left hip and was unable to walk with weight bearing; his left lower limb was shortened and externally rotated, with normal blood flow and sensation in the affected limb. After the injury, the blood circulation and sensation of the affected limb were normal. The patient was admitted to the local hospital, and x-ray of the left hip revealed a left-femur fracture, proximal-femur shepherd's crook deformity, and a neck stem angle close to 90°. The proximal femur could be seen as a pseudojoint. The local hospital has a limited medical level, and it is suggested that patients be transferred to a higher-level hospital for further treatment. The patient was therefore transferred to our hospital by ambulance. The doctor on duty provided tibial tuberosity bone traction to maintain the line of force of the lower limb and stabilize the end of the fracture to reduce pain. On the second day after admission, routine preoperative examination, including orthopedic x-ray of both hips and of the tibiofibula, x-ray of both hips, CT of the chest, and CT of the pelvis, were performed. A healthy femur, tibiofibula, sciatic bone, pubic bone, acetabulum, and ribs were observed. The patient was asked about his medical history. The patient was previously treated with surgery on his left femur in childhood, though the specific details of the surgery were not known due to the age of the patient. The details of the surgery are unknown. In adulthood, the left lower limb was shorter, and the patient walked with a limp on crutches without pain or discomfort (Figure 1).

**Figure 2:**
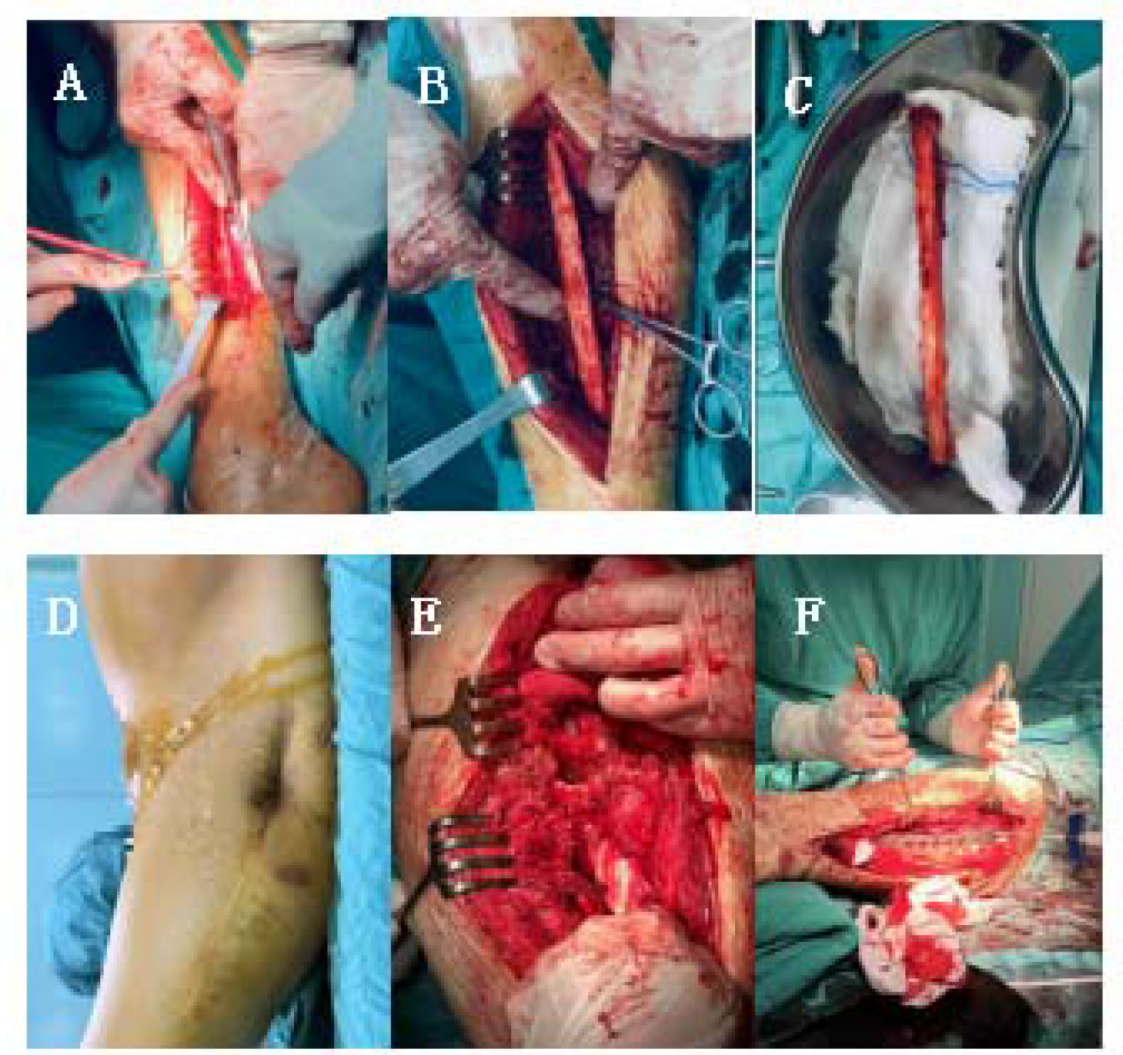
Intraoperative imaging **(A–F)**. **(A–C)** The fibula of the lower extremity on the same side was taken during the operation, with **(A)** length of about 12 cm **(D)** scar an local skin depression of the left hip were visible. **(E**,**F)** Femoral fracture was revealed during the operation, and pseudojoint activity was visible. During the operation, the steel plate was firmly fixed, and calcium sulfate liquid artificial bone was implanted in the bone defect to increase the stability of the screw.

Through perioperative treatment, contraindications to surgery were ruled out, and we communicated with the patient and his family about the disease and obtained consent for surgery. The surgery was performed under general anesthesia. First take the ipsilateral lower limb long enough fibula, suture pressure bandage incision. The original surgical incision scar on the left hip was noted, with the incision approximately 30 cm long. This fully revealed the fracture end, and the fracture hematoma was cleared. Femur pseudoarticular activity was noted during the operation, and medullary drilling using a No. 7 drill bit was applied to expand to the size of a No.9 bit. The autologous large section of the fibula was implanted in the medullary cavity, proximal to the top of the rotator cuff. The femoral epicondylar locking plate was inverted and placed; 5 screws were screwed into the distal end, and 6 screws were screwed into the proximal end. Four screws were implanted into the fibula for fixation, and the proximal end of the femur was fixed with steel cables. The fracture end and the pseudojoint were stabilized by moving the hip joint during the operation, and liquid calcium sulfate bone was implanted into the void in the bone marrow cavity of the femur, which increased the holding power of the proximal screws. The fracture was anatomically reset and well positioned based on intraoperative fluoroscopy (Figure 2).

**Figure 3:**
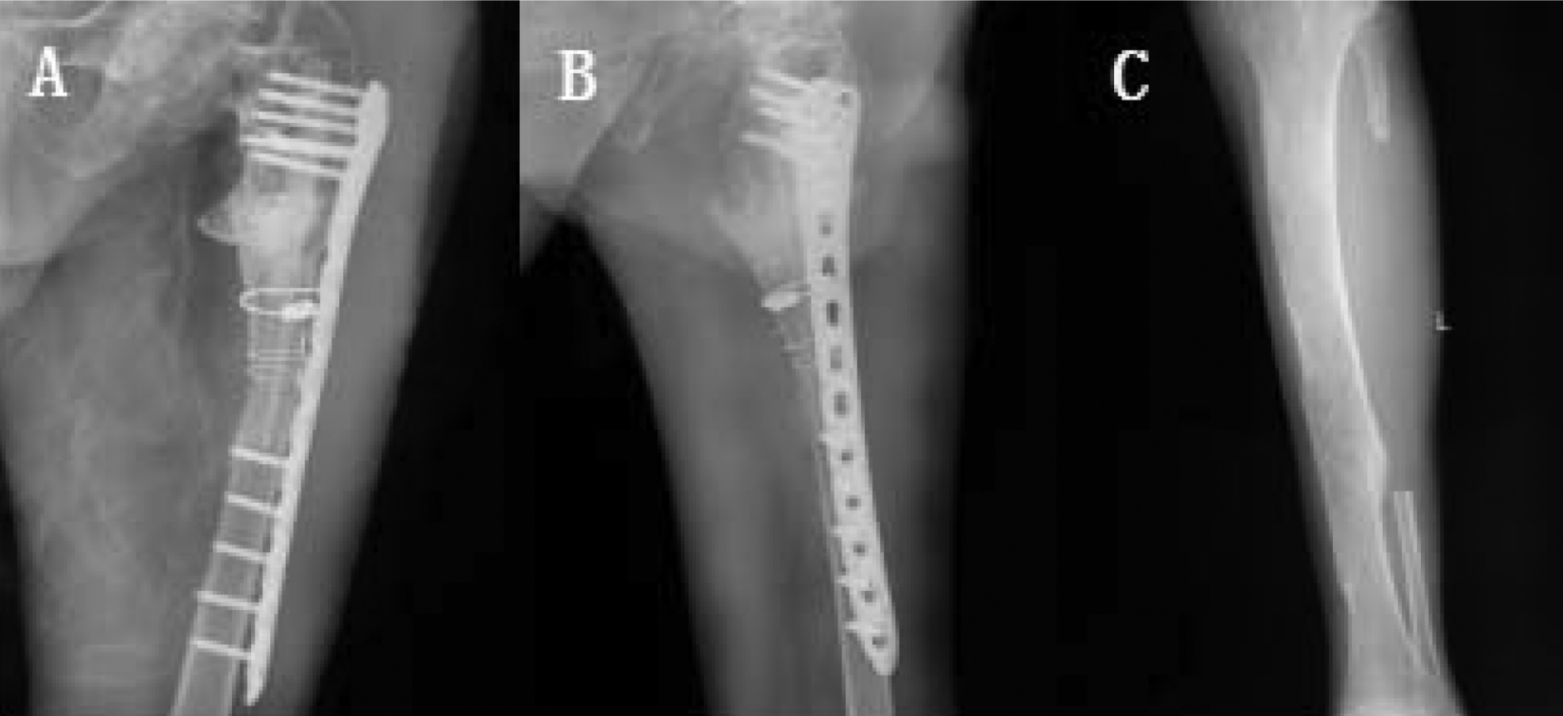
**(A)** Intraoperative fluoroscopy showing the fracture. Anatomically repositioned with internal fixation in good position. **(B)** A 12-month postoperative review image of front and side x-rays showing fracture healing, with fusion at the femoral pseudoarticulation and no loosening of the internal fixation. **(C)** A 12-month postoperative review of the left lower extremity with a 12-posterior x-ray of the fibula revealing no fracture of the tibia.

Postoperatively, the left lower limb was elevated with a lower-limb pad to promote swelling, and anticoagulation was routinely given to prevent lower-limb venous thrombosis. Active flexion and extension of the ankle and toe joints and passive flexion and extension of the knee joint were assessed. Postoperative x-ray of the left femur and left tibia was repeated at 1, 4, 8 and 12 weeks to understand the internal fixation of the plate and the healing of the fracture. At 6 months after the operation, x-ray of the left femur in the lateral position showed that the fracture end had healed, and the patient was allowed to walk with the aid of a walker. By the last follow-up at 12 months, the internal fixation was stable without loosening, the fracture end had completely healed, and the patient had resumed walking with a cane, as before the fracture. The patient had walked with crutches before the fracture and did not experience any discomfort in the left lower limb (Figure 3).

## Discussion

Fibrous dysplasia is a mosaic disease of bone, with bone replaced by fibro-osseous tissue or irregular trabeculae of woven bone intermixed with mature collagenous tissue (7). Although the pathogenesis of this disease is still controversial, it mainly involves GNAS gene mutations, abnormal chromosome structure and number, endocrine dysfunction, and abnormal bone development (8). Fibrous dysplasia may be characterized by monosclerosis (70%–80%), polyosclerosis (20%–30%), McCune-Albright syndrome (2%–3%), or Mazabraud syndrome, another manifestation of fibrous dysplasia, which is a very rare type of polyosclerosis. Intramuscular myxomas may occur alone or in combination (9). Fibrous dysplasia is slow, often occurs in adolescents, and can progress slowly throughout life. Although osteofibrous dysplasia is a benign lesion, it is generally assumed that FD accounts for 2.5%–5% of all benign bone lesions (10). Moreover, it has the potential for malignant transformation, in approximately 1% of all cases, and it has been reported in both monoosseous and multiosseous fibrous dysplasia (11, 12). The most common malignant changes involve sarcoma, fibrosarcoma, ductal carcinoma, and malignant fibroma. Patients who receive no treatment have poor prognosis, with a median survival of only 4 months (13).

Due to the osteogenic imperfection of bone with fibrous dysplasia, the stress-bearing capacity of bone is significantly decreased, and the proximal femur is the most concentrated part of the stress system and most frequently involved in FD (14). If the normal bone trabecular structure is replaced by abnormal fibrous tissue, the local bone anatomy may be damaged, reducing mechanical strength and leading to the potential risk of micropathological fractures. In addition, under the action of stress, the neck of the femur is curved, and the trunk angle of the neck is reduced, which induces hip varus deformity. Hip varus deformity further increases the stress load on the proximal femur; in some cases, fracture at the proximal femur where the stress is most concentrated occurs, eventually leading to deformity (15, 16). The corresponding clinical manifestations are limb shortening, claudication, joint pain, etc., which seriously affect the daily life of patients (17). To date, there are no effective drugs to correct osteogenic imperfecta with fibrous dysplasia, and surgical treatment is the only strategy for correcting the deformity and terminating the cycle of “stress depression-varus-pathological fracture”. FD patients often exhibit involvement of long limbs and craniofacial bones, and the surgical methods used for treating FD differ among different body parts; hence, there is no unified standard. Common surgical treatments include curettage, drilling, decompression, bone grafting and internal fixation. Curettage and bone grafting are the most commonly used surgical procedures. A high-speed grinding drill is used to remove the tumor, and allograft bone is used to fill the cavity, which can achieve satisfactory surgical results and functional recovery in the later stage (18, 19). For patients with relatively large lesions in the proximal femur and obvious shepherd's crook deformity, osteotomy and orthopedic internal fixation after bone grafting should be performed. Dynamic hip screw, hollow screw internal fixation, anatomical plate internal fixation, or intramedullary nail fixation can be selected according to the fixation method within the lesion range (20). Surgical treatment, such as intramedullary nailing + corrective osteotomy, is mostly used for children. For those with small-sized femurs, a modified humeral intramedullary nail is used, and when the femur grows to a sufficient size, it is replaced. Femoral intramedullary nailing also includes valgus osteotomy + lesion curettage + allogeneic bone graft + PHP fixation and valgus osteotomy combined with growing rods and tension band construct (21). For adults, this technique includes dynamic hip screw (DHS) or locking compression plate proximal screw (LCP) combined with intramedullary cortical support allogeneic bone (fibula) fixation and curettage + bone graft + intramedullary nail fixation. Some doctors also use an “angled-blade plate”, which considers that partially resorbed cortical bone is generally not suitable for locking the proximal screw of the intramedullary nail, and the angled-blade plate can be inserted with relative ease, thus providing adequate mechanical support (22).

Regarding the surgical plan, the patient was 44 years old, and according to preoperative imaging, the patient's acetabulum and proximal femur were damaged, and the bone cortex was thin; if total hip replacement was performed, the femoral prosthesis would be movable. The risk of periprosthetic fracture was very high, and there might be many future revision surgeries. An intramedullary femoral nailing protocol might be implemented to treat the patient's proximal-femur shepherd's crook deformity, but the neck stem angle was 90°s, and the proximal femur was empty, and the bone cortex was thin. Thus, the bone cortex could not hold screws to stabilize the end of the fracture, the proximal fracture would be unstable, and surgical failure would be high. Finally, we chose to use an allogeneic fibula graft and expand the bone marrow to open the femoral pseudoarticular joint. The femoral epicondylar locking plate was inverted, and the screw was implanted into the medullary cavity of the fibula. Nails were implanted into the medullary cavity of the fibula to stabilize it, and the proximal end of the femur was tied with steel cables to ensure adequate stabilization.

## Conclusions

Systemic multiple osteofibrous dysplasia accompanied by proximal-femur shepherd's crook deformity combined with femoral fracture and femoral pseudojoint formation is clinically uncommon. The choice of treatment should be based on the imaging and clinical characteristics of the patient, and the selection of an internal fixation plan should consider factors such as the lesion location, scope, nature and surrounding bone mass. This clinically rare condition needs to be further reported and summarized in more cases in the future.

## Data Availability

The original contributions presented in the study are included in the article/Supplementary Material, further inquiries can be directed to the corresponding author.
